# Functional connectivity in cortical regions in dementia with Lewy bodies and Alzheimer's disease

**DOI:** 10.1093/brain/awr327

**Published:** 2011-12-20

**Authors:** Eva R. Kenny, Andrew M. Blamire, Michael J. Firbank, John T. O'Brien

**Affiliations:** 1 Institute for Ageing and Health, Newcastle University, Campus for Ageing and Vitality, Newcastle upon Tyne, NE4 5PL, UK; 2 Institute of Cellular Medicine, Newcastle University, Newcastle upon Tyne, NE1 7RU, UK

**Keywords:** dementia with Lewy bodies, functional magnetic resonance imaging, resting-state, functional connectivity

## Abstract

Using resting-state functional magnetic resonance imaging, spontaneous low-frequency fluctuations in the blood oxygenation level-dependent signal were measured to investigate connectivity between key brain regions hypothesized to be differentially affected in dementia with Lewy bodies compared with Alzheimer's disease and healthy controls. These included connections of the hippocampus, because of its role in learning, and parietal and occipital areas involved in memory, attention and visual processing. Connectivity was investigated in 47 subjects aged 60 years and over: 15 subjects with dementia with Lewy bodies, 16 subjects with Alzheimer's disease and 16 control subjects. Subjects were scanned using a 3 Tesla magnetic resonance imaging system. The mean blood oxygenation level-dependent signal time series was extracted from seed regions in the hippocampus, posterior cingulate cortex, precuneus and primary visual cortex and correlated with all other brain voxels to determine functional connectivity. Both subjects with dementia with Lewy bodies and Alzheimer's disease showed greater connectivity than control subjects. Compared with controls, the dementia with Lewy bodies group had greater connectivity between the right posterior cingulate cortex and other brain areas. In dementia with Lewy bodies, there were no significant differences in hippocampal connectivity compared with controls, but in Alzheimer's disease left hippocampal connectivity was greater compared with controls. There were no significant differences between groups for precuneus or primary visual cortex connectivity. No seed regions showed significantly less connectivity in subjects with dementia with Lewy bodies or Alzheimer's disease compared with controls. We found greater connectivity with the posterior cingulate in dementia with Lewy bodies and with the hippocampus in Alzheimer's disease. Consistent with the known relative preservation of memory in dementia with Lewy bodies compared with Alzheimer's disease, hippocampal connectivity was not found to be greater in dementia with Lewy bodies. Importantly, while metabolic imaging shows functional change in primary visual cortex in dementia with Lewy bodies, which is hypothesized to account for visual hallucinations, we found connectivity with this region to be unaffected. This implicates areas beyond visual sensory input level in the visual symptoms and visual–perceptual dysfunction seen in dementia with Lewy bodies.

## Introduction

Dementia with Lewy bodies is the second most common form of neurodegenerative dementia (20% of cases at autopsy) after Alzheimer's disease (50–60% of cases at autopsy) (McKeith *et al*., 1996). The cognitive profile varies between the disorders; dementia with Lewy bodies is associated with greater deficits on attentional and visuo-perceptual tasks (Calderon *et al*., 2001; Collerton *et al*., 2003), whereas Alzheimer's disease is characterized by greater impairments in memory (Calderon *et al*., 2001; Ferman *et al*., 2006). However, clinical symptoms can greatly overlap, especially in the early stages, meaning differential diagnosis is often difficult.

Neuroimaging studies have provided important information on structural brain differences between dementia with Lewy bodies and Alzheimer's disease, which help to explain the varying symptom profiles, though there are still relatively few studies in dementia with Lewy bodies. MRI studies have shown that Alzheimer's disease is characterized by atrophy of medial temporal lobe structures that are the site of early pathological change, specifically the hippocampus and entorhinal cortex (Jack *et al*., 1997, 2002; Killiany *et al*., 2000; Du *et al*., 2001; Kenny *et al*., 2008; Firbank *et al*., 2010). In contrast, there is relative preservation of the hippocampus in dementia with Lewy bodies (Barber *et al*., 1999, 2000; Ballmaier *et al*., 2004; Burton *et al*., 2009).

Single photon emission computed tomography and PET studies report decreased activity in the posterior cingulate cortex in Alzheimer's disease (Minoshima *et al*., 1997; Johnson *et al*., 1998). These findings support the view that a distributed brain network (comprising the posterior cingulate and medial temporal lobe structures), which is involved in memory is affected in early stage Alzheimer's disease, with posterior cingulate changes associated with degeneration in distant but connected areas, e.g. the entorhinal cortex (Hirao *et al*., 2006). Single photon emission computed tomography studies have also shown decreased cerebral blood flow in the parietal and frontal lobes in dementia with Lewy bodies and Alzheimer's disease compared with controls, and greater deficits in occipital (visual areas) and posterior parietal cortices (precuneus) in dementia with Lewy bodies, whereas in Alzheimer's disease temporal regions are more affected (Colloby *et al*., 2002).

Functional MRI has some advantages over single photon emission computed tomography and PET, being non-invasive and offering higher spatial resolution. The most common functional MRI method uses the blood oxygen level-dependent response to map brain activity, based on the differential magnetic properties of oxygenated and deoxygenated haemoglobin and the coupling of oxygenated blood flow and neuronal activity (Ogawa *et al*., 1990). However, it should be noted, that the relationship between magnetic resonance signal and the physiological mechanisms underlying the blood oxygen level-dependent signal is not yet fully understood.

Many functional MRI studies focus on brain regions showing neuronal activity when an experimental task is performed, but spontaneous low-frequency fluctuations (at <0.10 Hz) in the blood oxygen level-dependent signal have been observed when a subject rests in the scanner (Biswal *et al*., 1995; Lowe *et al*., 1998; Cordes *et al*., 2000, 2001). Spatially distributed patterns of highly correlated spontaneous low-frequency fluctuations have been described and these have subsequently been termed resting-state networks, of which at least eight have been identified and are thought to represent functional connectivity (Beckmann *et al*., 2005; Damoiseaux *et al*., 2006; De Luca *et al*., 2006; Fox and Raichle, 2007).

Resting-state functional MRI studies in Alzheimer's disease have generally focused on examining connectivity with brain regions previously implicated in the disorder, for example the hippocampus or posterior cingulate cortex. There has only been one previous study investigating connectivity in dementia with Lewy bodies, which focused exclusively on precuneus connectivity and considered the whole structure as a single region, independent of laterality. Galvin *et al*. (2011) used a combined bilateral seed that showed increased connectivity with the putamen and inferior parietal cortex and decreased connectivity with the medial prefrontal cortex, frontoparietal operculum and primary visual cortex. The current study investigated and compared functional connectivity in dementia with Lewy bodies and Alzheimer's disease by correlating the time series of spontaneous low-frequency fluctuations in the blood oxygen level-dependent signal in the hippocampus, posterior cingulate cortex, precuneus and primary visual cortex with all other brain voxels to address three main hypotheses.

First, we hypothesized that functional connectivity between the hippocampus and cortical projection areas would be affected in Alzheimer's disease but preserved in dementia with Lewy bodies compared with control subjects. This hypothesis was based on neuropathological studies showing the hippocampus to be one of the first regions to be affected by neurofibrillary tangles and neuritic plaques in early Alzheimer's disease (Jack *et al*., 1992), and MRI studies showing that hippocampal volume is relatively preserved in dementia with Lewy bodies compared with Alzheimer's disease (Barber *et al*., 2000; Burton *et al*., 2009). Second, we hypothesized that functional connectivity to the posterior cingulate cortex and precuneus would be affected in both dementia groups compared with controls. The posterior cingulate and precuneus form part of a resting-state network, the default mode network, which is involved in attending to environmental stimuli (Raichle *et al*., 2001) and has previously been shown to be affected in Alzheimer's disease (Greicius *et al*., 2004; Zang *et al*., 2004; Zhang *et al*., 2009). Finally, as a control analysis we hypothesized that connectivity with the primary visual cortex would be unaffected across subject groups, as vision is not impaired in Alzheimer's disease and in dementia with Lewy bodies complex visual hallucinations may be associated with dysfunction in higher association cortex (Collerton *et al*., 2005). Since previous functional connectivity studies have shown increased left and decreased right hippocampal connectivity in Alzheimer's disease (Wang *et al*., 2006) all hypotheses were tested separately in each hemisphere.

## Materials and methods

### Subjects and assessment

The study involved 47 subjects, aged >60 years; 15 dementia with Lewy bodies, 16 Alzheimer's disease and 16 similarly aged healthy control subjects. Subjects with dementia with Lewy bodies and Alzheimer's disease were recruited from clinical Old Age Psychiatry, Geriatric Medicine and Neurology outpatient services and controls were recruited by local advertisement or were partners of subjects. The study was approved by the local ethics committee and all subjects gave signed informed consent for participation, according to the Declaration of Helsinki. Subjects with Alzheimer's disease fulfilled National Institute of Neurological and Communicative Diseases and Stroke/Alzheimer's Disease and Related Disorders Association (NINCDS/ADRDA) criteria for probable Alzheimer's disease (McKhann *et al*., 1984) and patients with dementia with Lewy bodies met consensus criteria for probable dementia with Lewy bodies, including the presence of two or more core features (fluctuating cognition, visual hallucinations and/or parkinsonism) (McKeith *et al*., 1996, 2005). Diagnoses were made by consensus between two experienced clinicians, a method that has previously been validated against autopsy diagnosis (McKeith *et al*., 2000). Nine subjects with dementia with Lewy bodies had a ^123^I-labelled *N*-(3-fluoropropyl)-2β-carbomethoxy-3β-(4-iodophenyl) nortropane (^123^I-FP-CIT) single photon emission computed tomography scan and all demonstrated reduced dopamine transporter uptake in the basal ganglia consistent with their diagnosis.

Detailed physical, neurological and neuropsychiatric examinations were carried out. Cognitive and neuropsychiatric examinations included Mini-Mental State Examination (Folstein *et al*., 1975), Cambridge Cognitive Examination (Roth *et al*., 1986), Geriatric Depression Score (Sheikh and Yesavage, 1986), Neuropsychiatric Inventory (Cummings *et al*., 1994), Clinical Assessment of Fluctuation Scale (Walker *et al*., 2000) and motor subsection of the Unified Parkinson's Disease Rating Scale (UPDRS III) (Fahn *et al*., 1987). Exclusion criteria for the study included severe concurrent illness (apart from dementia in the dementia with Lewy bodies and Alzheimer's disease groups), presence of space occupying lesions on MRI, stroke history and or presence of infarcts on scans, and contraindications to MRI. Control subjects had no history of psychiatric illnesses.

### Imaging

Subjects were scanned using a 3 Tesla MRI system (Intera Achieva scanner, Philips Medical System). An eight-channel head coil was used to collect resting-state functional MRI scans using a gradient-echo echo-planar imaging sequence. Subjects were asked to lie still, relax and keep their eyes closed but not to fall asleep. The timings and parameters used were similar to those used in previous resting-state studies (De Luca *et al*., 2006); 25 contiguous axial slices, 128 volumes, anterior-posterior acquisition, in-plane resolution = 2 × 2 mm, slice thickness = 6 mm, repetition time = 3000 ms, echo time = 40 ms, field of view = 260 × 260 mm, acquisition time = 6.65 min.

### Analysis

Analysis used the methods described by Fox *et al.* (2005), which involved removing all non-brain structures, correcting for involuntary head motion, spatial smoothing (6 mm full-width at half maximum), temporal band-pass filtering between 0.009 and 0.08 Hz to remove low-frequency drift and high frequency noise and registration to a study-specific functional brain template that was created to take into account the greater brain atrophy of the elderly study subjects. All analysis was performed using standard tools from the FMRIB Software Library package (Smith *et al*., 2004; FMRIB Analysis Group, 2007).

**Figure 1:**
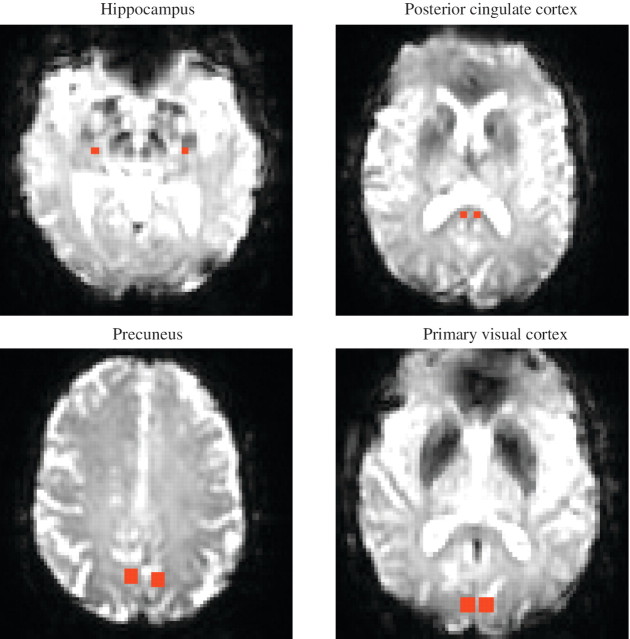
Seed regions overlaid on the functional images. Axial view of seed regions placed in the left and right hemisphere in a healthy control subject.

Seed regions were placed manually in each subject in the left and right hippocampi and posterior cingulate cortices (2 × 2 voxels), and precuneus and primary visual cortices (4 × 4 voxels) (Fig. 1). The mean blood oxygen level-dependent signal time series was extracted from each seed region and used as the model response function in a general linear model analysis. This enabled the measurement of functional connectivity assessed by the degree of correlation between the spontaneous low-frequency fluctuations in the seed region with signal variations in all other brain voxels (Woolrich *et al*., 2001). To ensure any non-neuronal fluctuations in the data did not confound analysis, time series from seeds placed in the white matter and CSF were included in the linear regression analysis as covariates of no interest together with a whole brain mask to remove any affects of global fluctuations (Fox *et al*., 2005).

A three group comparison (dementia with Lewy bodies, Alzheimer's disease and control groups) was carried out to investigate whether there were significant differences in connectivity between groups for each of the seed regions, by comparing their data on a voxel-by-voxel basis (Beckmann *et al*., 2003; Woolrich *et al*., 2004). *z*-Statistic (Gaussianized *T*/*F*) images were thresholded using clusters of pixels determined by *z* > 2.3 and *P* ≤ 0.05 (corrected cluster significance threshold) (Worsley, 2001). The peak connectivity cluster coordinates were converted from Montreal Neurological Index (MNI) space to Talairach space (Talairach and Tournoux, 1988) using the GingerALE software (Lancaster *et al*., 2007) and entered into Talairach Client, which assigns Talairach labels (hemisphere, lobe, gyrus, tissue and cell type) (Lancaster *et al*., 2000).

Statistical analysis was performed using Statistical Package for Social Sciences (version 15.0.1) (SPSS for Windows, 2006). One-way analysis of variance (ANOVA) was used to compare demographic factors across groups and the independent-samples *t*-test for comparisons between groups. The *P*-value for statistical significance was ≤0.05.

## Results

### Demographics

Table 1 shows the clinical characteristics of the study subjects. Groups were comparable for age (*P =* 0.294, *df =* 2, *F =* 1.26) and sex (*P =* 0.466, χ^2^ = 0.532, *df* = 1). As expected, controls had significantly higher scores on cognitive tests (Mini-Mental State Examination and Cambridge Cognitive Examination) and lower scores on measures of motor features (UPDRS) and depression (Geriatric Depression Score), compared with subjects with dementia with Lewy bodies and Alzheimer's disease. There were no significant differences between the groups with dementia with Lewy bodies and Alzheimer's disease in age at onset of dementia, duration of dementia, Mini-Mental State Examination or total Cambridge Cognitive Examination scores. Consistent with the known preservation of memory in dementia with Lewy bodies, subjects had significantly higher scores than Alzheimer's disease on the Cambridge Cognitive Examination memory subscore (*P =* 0.022), though significantly lower than control subjects (*P <* 0.001). UPDRS, Neuropsychiatric Inventory, Clinical Assessment of Fluctuation Scale and Geriatric Depression Score scores were significantly higher in subjects with dementia with Lewy bodies compared with Alzheimer's disease, indicating greater severity in dementia with Lewy bodies of the motor features of parkinsonism (*P <* 0.001), neuropsychiatric disturbances (*P =* 0.002), fluctuating cognition (*P =* 0.006) and depressive symptoms (*P =* 0.001). All of these differences were expected given the known symptom profile of dementia with Lewy bodies compared with Alzheimer's disease.

**Table 1:** Demographic and neuropsychological data of study subjects

Demographic and neuropsychological data	Dementia with Lewy bodies	Alzheimer's disease	Controls	*P*-value ANOVA
*n*	15	16	16	
Age (years)	80.6 ± 6.0	77.3 ± 8.9	76.3 ± 8.3	0.294
Age at onset of dementia (years)	77.2 ± 6.7	73.9 ± 8.9	n/a	0.256
Duration of dementia (months)	40.2 ± 20.3	40.4 ± 24.8	n/a	0.981
Mini-Mental State Examination	19.5 ± 4.2	21.1 ± 3.5	28.6 ± 1.3	<0.001*
Cambridge Cognitive Examination Total	69.0 ± 12.9	68.9 ± 11.4	96.9 ± 3.5	<0.001*
Cambridge Cognitive Examination Memory	16.0 ± 4.9	12.1 ± 4.0	24.1 ± 2.1	<0.001*
UPDRS	22.1 ± 11.9	6.1 ± 4.4	2.7 ± 3.6	<0.001*
Neuropsychiatric Inventory	23.1 ± 11.5	8.5 ± 11.8	n/a	0.002*
Clinical Assessment of Fluctuation Scale	6.7 ± 5.3	1.6 ± 3.4	n/a	0.006*
Geriatric Depression Score	7.1 ± 3.3	3.4 ± 2.4	1.3 ± 1.4	<0.001*

The *P*-values were calculated using the Independent-Samples *t*-test: Cambridge Cognitive Examination Memory: dementia with Lewy bodies > Alzheimer's disease (*P =* 0.022, *df =* 29, *t =* −2.42); UPDRS: Controls < Alzheimer's disease (*P =* 0.023, *df =* 30, *t =* −2.40) (i.e. subjects with Alzheimer's disease performed significantly worse); Neuropsychiatric Inventory: dementia with Lewy bodies > Alzheimer's disease (*P =* 0.002, *df =* 27, *t =* −3.38) (i.e. subjects with dementia with Lewy bodies performed significantly worse); Clinical Assessment of Fluctuation Scale: dementia with Lewy bodies > Alzheimer's disease (*P =* 0.01, *df =* 24, *t =* −2.8) (i.e. subjects with dementia with Lewy bodies performed significantly worse); and Geriatric Depression Score: Controls < Alzheimer's disease (*P =* 0.006, *df =* 30, *t =* −2.95). Alzheimer's disease < dementia with Lewy bodies (*P =* 0.001, *df =* 29, *t =* −3.60). Controls < dementia with Lewy bodies (*P* < 0.001, *df =* 29, *t =* −6.39).

Values expressed as mean ± standard deviation.

n/a = not applicable; *P < 0.05.

At the time of study, 24 subjects were taking acetyl cholinesterase inhibitors; 14 subjects with Alzheimer's disease (donepezil, *n =* 9 and galantamine, *n =* 5) and 10 subjects with dementia with Lewy bodies (donepezil, *n =* 5; galantamine, *n =* 4; and rivastigmine, *n =* 1). Eight subjects (six with dementia with Lewy bodies and two with Alzheimer's disease) were taking anti-depressants (citalopram, mirtazapine, trazodone, venlafaxine or paroxetine), and one subject with dementia with Lewy bodies was taking a benzodiazepine (zopliclone) as a hypnotic.

### Functional connectivity

**Figure 2:**
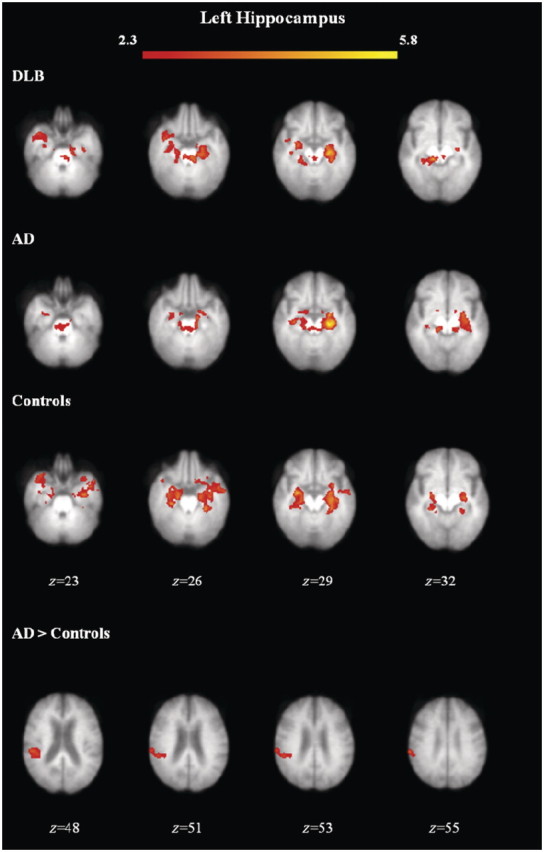
Functional connectivity maps for group means and group differences with the left hippocampus. Axial view of brain regions showing significant connectivity (*z*-statistic > 2.3, *P <* 0.05) overlaid on the mean structural scan of all study subjects. AD = Alzheimer's disease; DLB = dementia with Lewy bodies.

Functional connectivity maps for the left hippocampus in each group are shown in Fig. 2, with the brain regions and respective coordinates showing significant functional connectivity with the left and right hippocampi shown in Table 2. Formal statistical comparison of group results showed that there were only significant differences between Alzheimer's disease and controls for the left hippocampus. Connectivity was greater between the left hippocampus and right insula and inferior parietal regions in subjects with Alzheimer's disease compared with controls (Fig. 2 and Table 2). There were no other significant differences between groups for the left or right hippocampus.

**Table 2:** Brain regions showing significant functional connectivity with hippocampal seed regions

	Connectivity with left hippocampus		Connectivity with right hippocampus	
Brain region	Talairach coordinates	*Z*-score	Talairach Coordinates	*Z*-score
Dementia with Lewy bodies (mean)				
Parahippocampal (right)	–	–	25, −23, −14	5.98
Parahippocampal (left)	−23, −19, −14	4.92	−25, −32, −10	4.76
Culmen (right)	10, −29, −9	4.67	25, −31, −20	3.71
Culmen (left)	−8, −29, −20	3.77	−18, −32, −15	3.61
Superior temporal (left)	–	–	−30, 7, −28	3.73
Subcallosal (right)	–	–	7, 5, −11	3.58
Alzheimer's disease (mean)				
Parahippocampal (right)	32, −17, −13	3.73	23, −23, −14	5.19
Parahippocampal (left)	−23, −21, −14	5.86	−19, −13, −14	3.92
Middle temporal (right)	35, −1, −28	4.36	40, 1, −27	4.20
Culmen (right)	14, −29, −9	3.66	–	–
Culmen (left)	–	–	−16, −39, −16	4.46
Uncus (right)	–	–	20, −12, −29	4.38
Controls (mean)				
Parahippocampal (right)	21, −21, −14	4.16	21, −19, −13	5.78
Parahippocampal (left)	−27, −17, −14	4.15	−19, −25, −9	3.74
Culmen (right)	–	–	6, −40, −5	4.38
Culmen (left)	−27, −31, −21	4.22	−25, −34, −16	3.55
Uncus (left)	−34, −8, −24	4.08	–	–
Middle temporal (left)	−43, 5, −28	3.92	−39, 3, −29	3.27
Amygdala (left)	–	–	−23, −10, −8	4.16
Putamen (left)	–	–	−27, −10, −8	4.12
Superior temporal (left)	–	–	−34, 12, −22	3.48
Lingual (left)	–	–	−10, −54, 4	2.64
Dementia with Lewy bodies > Alzheimer's disease	NS differences		NS differences	
Dementia with Lewy bodies > Controls	NS differences		NS differences	
Alzheimer's disease > dementia with Lewy bodies	NS differences		NS differences	
Alzheimer's disease > Controls			NS differences	
Inferior parietal (right)	49, −32, 24	3.52		
Insula (right)	39, −38, 23	3.23
Controls > dementia with Lewy bodies	NS differences		NS differences	
Controls > Alzheimer's disease	NS differences		NS differences	

*Z*–Statistic images were thresholded using clusters determined by *z >* 2.3 and a corrected cluster significance of *P <* 0.05.

NS = not significant.

**Figure 3:**
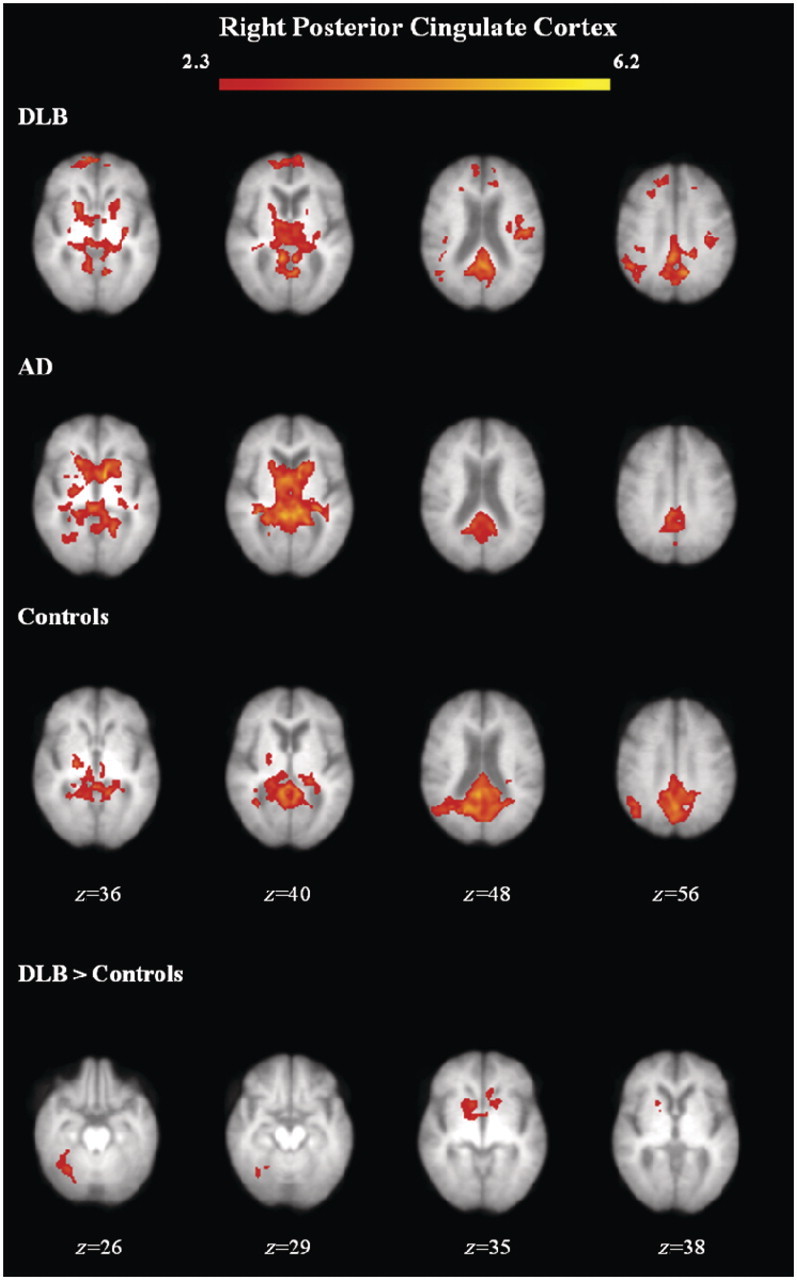
Functional connectivity maps for group means and group differences with the right posterior cingulate cortex. Axial view of brain regions showing significant connectivity (*z*-statistic > 2.3, *P <* 0.05) overlaid on the mean structural scan of all study subjects. AD = Alzheimer's disease; DLB = dementia with Lewy bodies.

Figure 3 shows the group mean connectivity maps with the right posterior cingulate cortex seed, with the brain regions and their coordinates showing significant functional connectivity; the left and right posterior cingulate seeds are shown in Table 3. Inspection of the patterns of connectivity in the group mean data showed that in control subjects, connectivity was confined mainly to posterior regions, while in both subjects with dementia with Lewy bodies and Alzheimer's disease, additional connectivity was observed with the insula, thalamus and/or caudate. Group statistical comparison for the posterior cingulate cortex showed greater connectivity in the group with dementia with Lewy bodies compared with controls between the right posterior cingulate and limbic (left anterior cingulate), sublobar (right globus pallidus), anterior lobe (right culmen) and posterior lobe (right cerebellar tonsil) (Fig. 3 and Table 3). There were no other significant differences between groups for the left or right posterior cingulate cortex.

**Table 3:** Brain regions showing significant functional connectivity with posterior cingulate cortex seed regions

	Connectivity with left posterior cingulate		Connectivity with right posterior cingulate	
Brain region	Talairach Coordinates	*Z*-score	Talairach Coordinates	*Z*-score
Dementia with Lewy bodies (mean)				
Posterior cingulate (right)	–	–	8, −45, 6	4.65
Posterior cingulate (left)	0, −52, 16	4.77	0, −52, 16	5.10
Lingual (left)	−5, −60, 4	4.68	–	–
Thalamus (right)	1, −27, 7	4.33	–	–
Culmen (right)	4, −41, 0	4.27	–	–
Precuneus (left)	0, −48, 32	4.24	−11, −63, 31	4.51
Uvula of vermis (right)	5, −60, −34	3.96	–	–
Medial frontal (right)	8, 38, 35	3.63	8, 58, 10	3.66
Cerebellar tonsil (right)	11, −50, −44	3.49	12, −48, −43	4.51
Cerebellar tonsil (left)	−23, −45, −38	3.57	–	–
Nodule (right)	1, −57, −28	3.56	–	–
Postcentral (left)	−35, −27, 28	3.53	−37, −28, 28	3.85
Cingulate (left)	−20, −38, 33	3.34	–	–
Superior frontal (right)	15, 18, 50	3.04	–	–
Superior temporal (right)	35, −74, 30	3.04	–	–
Insula (left)	−42, −19, 23	3.00	−44, −19, 23	3.96
Sub-gyral (right)	–	–	34, 44, −2	4.45
Middle frontal (right)	–	–	23, 23, 34	3.67
Inferior parietal (left)	–	–	−42, −32, 44	3.29
Alzheimer's disease (mean)				
Posterior cingulate (right)	1, −50, 16	5.59	4, −42, 11	5.27
Posterior cingulate (left)	–	–	−3, −46, 11	5.03
Cerebellar tonsil (left)	−10, −53, −33	4.76	–	–
Pyramis (right)	1, −65, −23	4.53	–	–
Declive (right)	6, −62, −18	4.19	–	–
Declive (left)	−10, −73, −19	4.50	–	–
Nodule (right)	1, −59, −28	4.40	–	–
Precuneus (left)	0, −46, 38	4.25	–	–
Thalamus (right)	3, −19, 8	4.09	–	–
Thalamus (left)	−10, −30, 7	4.04	–	–
Caudate head (left)	–	–	−8, 2, 4	5.12
Controls (mean)				
Posterior cingulate (right)	2, −48, 16	4.76	4, −46, 11	5.95
Posterior cingulate (left)	−3, −46, 16	5.41	−7, −48, 10	5.58
Cingulate (right)	–	–	6, −49, 27	5.08
Cuneus (left)	−3, −75, 19	5.57	–	–
Precuneus (right)	–	–	13, −60, 26	4.86
Precuneus (left)	–	–	−2, −63, 31	4.52
Globus pallidus (left)	−10, 1, −1	4.18	–	–
Caudate tail (right)	32, −44, 11	4.12	–	–
Cerebellar tonsil (left)	−3, −53, −33	3.78	−25, −62, −34	3.04
Pyramis (right)	12, −72, −29	3.65	–	–
Pyramis (left)	−18, −66, −29	3.51	−14, −68, −29	3.19
Inferior semi lunar (left)	−27, −67, −35	3.53	−23, −65, −40	3.44
Nodule (left)	−1, −59, −23	3.49	–	–
Parahippocampal (right)	–	–	32, −55, 10	4.44
Dementia with Lewy bodies > Alzheimer's disease	NS differences		NS differences	
Dementia with Lewy bodies > Controls	NS differences			
Cerebellar tonsil (right)			25, −43, −37	3.85
Culmen (right)			29, −56, −22	3.36
Anterior cingulate (left)			−3, 1, −1	2.91
Globus pallidus (right)			16, 2, −1	2.82
Alzheimer's disease > dementia with Lewy bodies	NS differences		NS differences	
Alzheimer's disease > Controls	NS differences		NS differences	
Controls > dementia with Lewy bodies	NS differences		NS differences	
Controls > Alzheimer's disease	NS differences		NS differences	

*Z*-Statistic images were thresholded using clusters determined by *z >* 2.3 and a corrected cluster significance of *P <* 0.05.

NS = not significant.

Supplementary Table 1 shows the regions of significant connectivity with the precuneus within each subject group. Voxel-wise group comparisons showed that there were no significant differences between groups for precuneus functional connectivity. The regions of significant connectivity with the primary visual cortex within each subject group are shown in Supplementary Table 2. Similar to the precuneus findings, there were no significant differences in functional connectivity between groups with the left or right primary visual cortex seeds.

In summary, subjects with dementia with Lewy bodies showed greater connectivity than controls with the right posterior cingulate cortex and subjects with Alzheimer's disease showed greater connectivity than controls with the left hippocampus. No brain regions showed lower connectivity in subjects with dementia with Lewy bodies or Alzheimer's disease compared with controls, and there were no significant differences in connectivity between the dementia groups.

## Discussion

The present resting-state functional MRI study investigated functional connectivity in subjects with dementia with Lewy bodies compared with Alzheimer's disease and control subjects. The main findings were greater functional connectivity between seed region(s) and other cortical and subcortical areas in subjects with dementia with Lewy bodies and Alzheimer's disease compared with controls. Connectivity with the right posterior cingulate cortex was greater in subjects with dementia with Lewy bodies compared with controls, while the left hippocampus had greater connectivity in subjects with Alzheimer's disease compared with controls. For the precuneus and primary visual cortex seeds, there were no significant differences in connectivity between groups. No brain regions showed lower connectivity in subjects with dementia with Lewy bodies or Alzheimer's disease compared with controls.

Resting-state functional MRI studies have been performed in subjects with Alzheimer's disease by a number of groups and have reported varying, and occasionally opposing findings, with some reporting their Alzheimer's disease cohort characterized by increased connectivity (Wang *et al*., 2007; Zhang *et al*., 2010), whereas others have reported decreased connectivity (Li *et al*., 2002; Greicius *et al*., 2004). Dementia with Lewy bodies has only been investigated in a single study that focused on the bilateral precuneus and reported both increased connectivity with the putamen and inferior parietal cortex and decreased connectivity between bilateral precuneus and the medial prefrontal cortex, frontoparietal operculum and primary visual cortex (Galvin *et al*., 2011). This study examined connectivity in a wider network of key brain structures known to be structurally, metabolically or functionally altered in subjects with dementia with Lewy bodies and Alzheimer's disease.

### Hippocampal connectivity

Intuitively, it might be expected that the characteristic atrophy of medial temporal lobe structures in Alzheimer's disease would be associated with decreased connectivity and this has been reported in several studies (Greicius *et al*., 2004; Allen *et al*., 2007). However, we observed greater connectivity between the left hippocampus and right insula and inferior parietal regions in Alzheimer's disease compared with control subjects. Similarly, other groups have reported increased connectivity between left hippocampus and right hemispheric regions in Alzheimer's disease compared with controls, though in the study by Wang *et al*. (2006) it was with prefrontal regions. Increases in short distance connections have also been reported in Alzheimer's disease in regions including the hippocampus (Yao *et al*., 2010) and a study of carriers of the apolipoprotein ε4 allele (a strong genetic risk factor for Alzheimer's disease) showed changes in similar regions to the subjects with Alzheimer's disease in our study; increased activity in insular and parietal regions compared with non-carriers (Westlye *et al*., 2011). Interestingly, an episodic memory study showed increased hippocampal activity in subjects who had less severe mild cognitive impairment (a transition stage between normal ageing and Alzheimer's disease) compared with controls, whereas subjects who had more severe mild cognitive impairment and mild Alzheimer's disease showed decreased hippocampal activity (Celone *et al*., 2006). These connectivity findings support the compensatory recruitment hypothesis, which proposes that connectivity (e.g. hippocampal) may initially increase in the earlier stages of disease with new brain areas being recruited to compensate for loss of others, before activity is decreased later in the course of the disease.

In further support of this hypothesis, our data found no significant differences in hippocampal connectivity between subjects with dementia with Lewy bodies and controls. A significant difference in hippocampal connectivity between subjects with dementia with Lewy bodies and Alzheimer's disease might have been expected, as the hippocampus shows greater atrophy in Alzheimer's disease than dementia with Lewy bodies (Barber *et al*., 2000), but no differences were found, suggesting that connectivity measures report of processes beyond structural degeneration.

### Posterior cingulate cortex connectivity

Subjects with dementia with Lewy bodies showed significantly greater connectivity than controls between the right posterior cingulate and the cerebellar tonsil, culmen, anterior cingulate and globus pallidus. The posterior cingulate cortex is a key region of the default mode network, which is proposed to be involved in ongoing/intrinsic brain activity, for example day dreaming and attending to environmental stimuli (Raichle *et al*., 2001; Raichle and Snyder, 2007). Attentional deficits are greater in subjects with dementia with Lewy bodies than Alzheimer's disease (Ballard *et al*., 2001) and therefore connectivity with this region may be expected to be more affected in dementia with Lewy bodies compared with subjects with Alzheimer's disease. Our findings, in part, support this as greater posterior cingulate connectivity compared with controls was specific to subjects with dementia with Lewy bodies, though we did not find significantly greater connectivity in subjects with dementia with Lewy bodies compared with subjects with Alzheimer's disease.

Previous resting-state studies in Alzheimer's disease have reported opposing findings in posterior cingulate connectivity of decreased connectivity (Greicius *et al*., 2004), or a combination of both increased and decreased connectivity with this area (Zhang *et al*., 2009). Similar to our study, a previous task-based investigation also showed posterior cingulate connectivity was affected in subjects with dementia with Lewy bodies and no significant differences between subjects with dementia with Lewy bodies and those with Alzheimer's disease, though in contrast to this study they also showed connectivity was affected in Alzheimer's disease (Sauer *et al*., 2006). The lack of significant findings in the Alzheimer's disease group in our study could be caused by a deficiency in compensatory recruitment with the posterior cingulate, possibly caused by Alzheimer's disease pathology in this region.

A study by Greicius *et al*. (2003) showed that connectivity analysis from a posterior cingulate cortex seed was able to identify the default mode network. In contrast, our study showed connectivity between the posterior cingulate and some (e.g. the precuneus), but not all of the default mode network regions across groups, suggesting altered functioning in this network. In support of our findings, a previous study of healthy elderly subjects showed decreased activity of the default mode network compared with younger control subjects, and specifically activity was decreased in the same regions where we showed no connectivity across groups (middle temporal and superior parietal regions) (Damoiseaux *et al*., 2008).

Interestingly, on visual inspection of our connectivity maps, right posterior cingulate connectivity in controls most closely matched the default mode network, whereas the group with Alzheimer's disease appeared to have the least connectivity in default mode regions. These findings may be linked with the theory that continuous activity of these regions in the resting-state results in an activity-dependent cascade, which is conducive to the formation of Alzheimer's disease pathology. This hypothesis is supported by the finding that there is a high concentration of plaques in these brain regions (Buckner *et al*., 2005).

### Precuneus connectivity

Precuneus connectivity was predicted to be affected in both dementia with Lewy bodies and Alzheimer's disease subjects’ compared with controls, and additionally more affected in patients with dementia with Lewy bodies than subjects with Alzheimer's disease. The precuneus is also a default mode network region (Raichle *et al*., 2001) and previous studies in Alzheimer's disease have reported reduced connectivity with this region (Zang *et al*., 2004; He *et al*., 2007; Zhang *et al*., 2009). A recent resting-state study investigated precuneus connectivity in dementia with Lewy bodies showing increased connectivity with the putamen and inferior parietal regions and decreased connectivity with medial prefrontal and primary visual cortices (Galvin *et al*., 2011). Using single photon emission computed tomography, significantly decreased perfusion in the precuneus in dementia with Lewy bodies compared with Alzheimer's disease has been reported (Colloby *et al*., 2002), therefore this may affect functional connectivity with this region.

In contrast to Galvin *et al*. (2011), we found no significant differences between groups for precuneus connectivity. The different findings between the studies could be explained by the different subject demographics and by differences in methodological approaches. Galvin *et al*. (2011) used standard coordinates to investigate bilateral precuneus connectivity whereas we placed left and right precuneus seeds individually for each subject and thus investigated unilateral connectivity. Our approach to manually place the seed regions aimed to take into account the greater atrophy in subjects with dementia whereas standard coordinates are based on younger healthy subjects. Additionally, the subjects with dementia with Lewy bodies in the Galvin *et al*. (2011) study were younger (mean of 72 years versus 81 years) and less cognitively impaired with a higher Mini-Mental State Examination score (25.0 versus 19.5). Although single photon emission computed tomography shows decreased perfusion in the precuneus in dementia with Lewy bodies (Colloby *et al*., 2002), it is not certain whether perfusion deficits affect functional connectivity. Similar to our results, task-based connectivity studies show the posterior cingulate is affected in dementia with Lewy bodies and Alzheimer's disease, but the precuneus was not shown to be affected in subjects with dementia with Lewy bodies or Alzheimer's disease (Sauer *et al*., 2006).

### Primary visual cortex connectivity

As hypothesized, there were no significant differences in connectivity with the primary visual cortex between any of the groups. Similarly, a recent functional MRI study showed no significant differences between subjects with dementia with Lewy bodies and controls in primary visual cortex activity in response to a visual stimulus task (Wood *et al*., 2011). Resting-state single photon emission computed tomography studies have shown decreased perfusion in dementia with Lewy bodies compared with Alzheimer's disease in the primary visual cortex (Colloby *et al*., 2002), which are thought to be associated with the core feature of visual hallucinations in dementia with Lewy bodies (McKeith *et al*., 1996). However, as mentioned previously, it is not certain whether perfusion deficits affect functional connectivity and additionally, it could be that in order for subjects with dementia with Lewy bodies to experience hallucinations their primary visual system needs to be intact.

Our findings support the perception and attention deficit model, which proposes that failings in sensory and attention functions are needed for visual hallucinations to occur. The failure to properly integrate sensory information (bottom–up) and prior expectation (top–down) in dementia with Lewy bodies is thought to be caused by cholinergic dysfunction, which affects higher association cortex, frontal cortex and ventral visual stream (Collerton *et al*., 2005), rather than due to changes in the primary visual cortex. The results in the Alzheimer's disease group of no significant differences in primary visual cortex connectivity match our hypothesis, as vision is not affected in Alzheimer's disease.

### Methodological differences between studies

As briefly mentioned above, the variation in methodology between studies may contribute to the differing results. For example, some studies using the posterior cingulate cortex as seed have used the whole region (Wang *et al*., 2007; Zhang *et al*., 2009), rather than placing four voxels within the region, whereas others have used standard coordinates to identify the seed region across all subjects and have investigated bilateral connectivity (Galvin *et al*., 2011), rather than manually placing the seed in each subject and investigating left and right connectivity separately as we did. Others have investigated whole lobe connectivity, e.g. parietal lobe (Wang *et al*., 2007) or connectivity within a brain structure only, e.g. the hippocampus (Li *et al*., 2002), rather than between a seed and all other brain voxels. Some studies have not included covariates of no interest to correct for non-neuronal fluctuations (Zhang *et al*., 2009) or temporal filtering (Li *et al*., 2002), which were used in this study. In contrast to the model-based approach used here, others have used model-free approaches of regional homogeneity (He *et al*., 2007) or independent component analysis (Greicius *et al*., 2004; Gili *et al*., 2011). Additionally, most of the previous resting-state studies in Alzheimer's disease and the study in dementia with Lewy bodies have been in younger subjects who had higher Mini-Mental State Examination scores, i.e. less cognitively impaired (He *et al*., 2007; Wang *et al*., 2007; Zhang *et al*., 2009; Galvin *et al*., 2011).

### Strengths and limitations

Strengths of this study were that the dementia with Lewy bodies and Alzheimer's disease groups were subject to full clinical and cognitive assessment and rigorous diagnosis. Groups were well matched for subject numbers, gender and age, with the dementia groups well matched for age at onset and duration of dementia, Mini-Mental State Examination and total Cambridge Cognitive Examination scores. This study used a model-based/hypothesis-driven analysis approach, enabling connectivity to be investigated between seeds and all other brain voxels. Seed regions were selected based on previous study findings showing these regions to be affected or unaffected in subjects with dementia with Lewy bodies and/or Alzheimer's disease compared with control subjects. In contrast, other studies have used model-free methods where no prior hypothesis is required and all connectivity is searched for. The seed regions investigated were placed manually in each subject, meaning the seed was less likely to be affected by atrophy differences between subjects. Resting-state studies benefit from simplicity of experimental design, no task has to be practised or performed, meaning they are advantageous in cognitively impaired patients for whom it may be more difficult to adhere to a task.

Limitations are that model-based techniques are biased by the choice of seed region and functional connectivities of interest can be missed if they do not show connectivity with the seed, though this bias can be reduced by investigating connectivity with all other voxels as was done in this study. Model-free methods do not require predefined seeds or a temporal model, but their lack of specificity means results can be hard to interpret. The number of subjects in each group was relatively small; with larger group sizes more significant differences between groups may have been observed. However, other resting-state functional MRI studies have had comparable numbers (Zhang *et al*., 2009), whereas others have had fewer subjects (Li *et al*., 2002; Gili *et al*., 2011).

Fluctuations unrelated to neuronal activity have been shown to influence resting-state data, for example cardiac (Chang and Glover, 2009) and respiratory (Wise *et al*., 2004; Birn *et al*., 2006) related signals. These fluctuations have been shown to correlate in grey matter brain regions and as they are non-neuronal in origin they are not of interest in functional connectivity analysis. In this study, spurious fluctuations were corrected for by carrying out additional preprocessing and regression steps that can be incorporated within the model-based analysis.

Resting-state is difficult to control, as between subjects it can vary greatly depending on how active a subjects’ brain is at rest and what the subject is thinking at the time of scanning. As there is no definitive diagnosis for dementia with Lewy bodies and Alzheimer's disease, subject groups cannot be confirmed until autopsy. Further limitations include the possible effects of medication on connectivity in the groups with dementia with Lewy bodies and Alzheimer's disease. A previous study showed that subjects with Alzheimer's disease taking memantine had increased precuneus activity compared with subjects with Alzheimer's disease not taking memantine (Lorenzi *et al*., 2011). In this study, none of the subjects with dementia were taking memantine and we showed no significant differences between groups for precuneus connectivity, however, the potential effects of other medications on connectivity cannot be ruled out. A longitudinal study of subjects before and after medication would be needed to address this.

Additionally, severity of neuropsychiatric symptoms could influence connectivity. For future work it would be important to compare whether subjects with less severe cognitive impairments (low Mini-Mental State Examination score) versus those with more severe cognitive symptoms (high Mini-Mental State Examination score) had significant differences in connectivity with memory structures, i.e. the hippocampus. Or, to investigate the effect on connectivity with the posterior cingulate in subjects with dementia with Lewy bodies with high levels of cognitive fluctuations versus those with low levels.

In conclusion, the main findings of this study were greater functional connectivity in dementia with Lewy bodies compared with control subjects with the posterior cingulate and in the subjects with Alzheimer's disease compared with controls with the hippocampus. Subjects with dementia with Lewy bodies or Alzheimer's disease did not show lower connectivity with any seed regions compared with control subjects. These findings may aid in greater understanding of the neurobiology of dementia with Lewy bodies and Alzheimer’s disease, which could assist in differentiation of the disorders that commonly show similar characteristics but require different management.

## Funding

This work was supported by a Medical Research Council Capacity Building Research Studentship, Alzheimer's Research UK (grant number ART-PPG2007A-2) and the UK NIHR Biomedical Research Centre for Ageing and Age-related disease award to the Newcastle upon Tyne Foundation Hospitals NHS Trust.

## Supplementary Material

Supplementary Data

## Abbreviations

UPDRS: Unified Parkinson's Disease Rating Scale
