# Supplementary material for: Functional connectivity in cortical regions in dementia with Lewy bodies and Alzheimer's disease
Source: Brain. 2011 Dec 20;135(2):569–81. doi: 10.1093/brain/awr327 (PMC3708629; doi:10.1093/brain/awr327)
Supplement: Supplementary Data [file supp_135_2_569__index.html]

Functional connectivity in cortical regions in dementia with Lewy bodies and Alzheimer's disease — Supplementary Data 

# Functional connectivity in cortical regions in dementia with Lewy bodies and Alzheimer's disease

## Supplementary Data

files

**Files in this Data Supplement:**

- Supplementary Data - docx file
