# Supplementary material for: Functional connectivity in cortical regions in dementia with Lewy bodies and Alzheimer's disease
Source: Brain. 2011 Dec 20;135(2):569–81. doi: 10.1093/brain/awr327 (PMC3708629; doi:10.1093/brain/awr327)
Supplement: Supplementary Data [file supp_awr327_Supplementary_material-Brain.docx]

**Table 1**: Brain Regions showing Significant Functional Connectivity with Precuneus Seed Regions

|  | **Connectivity with Left Precuneus** | |  | **Connectivity with Right Precuneus** | |
| --- | --- | --- | --- | --- | --- |
| **Brain Region** | **Talairach Coordinates** | **Z score** |  | **Talairach Coordinates** | **Z score** |
| **DLB Mean** |  |  |  |  |  |
| Precuneus (R) | 9, -63, 36 | 5.40 |  | 9, -65, 36 | 5.18 |
| Precuneus (L) | -5, -58, 42 | 4.58 |  | -7, -64, 47 | 5.30 |
| Middle Frontal (R) | 26, 7, 43 | 3.47 |  | - | - |
| Cingulate (R) | 17, 22, 28 | 2.98 |  | - | - |
|  |  |  |  |  |  |
| **AD Mean** |  |  |  |  |  |
| Precuneus (R) | 9, -63, 36 | 5.59 |  | 9, -65, 36 | 5.18 |
| Precuneus (L) | -5, -58, 42 | 5.54 |  | -7, -64, 47 | 5.40 |
| Middle Frontal (R) | 26, 7, 43 | 4.59 |  | - | - |
| Medial Frontal (R)  Cingulate (R) | 21, 24, 29  17, 22, 28 | 2.92  2.79 |  | -  - | -  - |
|  |  |  |  |  |  |
| **Controls Mean** |  |  |  |  |  |
| Precuneus (R)  Precuneus (L) | 4, -66, 41  -7, -68, 46 | 5.98  5.63 |  | 8, -54, 37  -7, -64, 47 | 6.52  6.20 |
| Cuneus (L) | -15, 82, 34 | 5.34 |  | - | - |
| Middle Frontal (L)  Claustrum (L)  Insula (L) | -  -  - | -  -  - |  | -25, 24, 33  -27, 16, 16  -33, 14, 16 | 4.95  3.46  2.87 |
|  |  |  |  |  |  |
| **DLB > AD**  **DLB > Controls**  **AD > DLB**  **AD > Controls**  **Controls > DLB**  **Controls > AD** | NS differences  NS differences  NS differences  NS differences  NS differences  NS differences |  |  | NS differences  NS differences  NS differences  NS differences  NS differences  NS differences |  |

Z Statistic images were thresholded using clusters determined by z>2.3 and a corrected cluster significance of p<0.05

**Table 2**: Brain Regions showing Significant Functional Connectivity with Primary Visual Cortex Seed Regions

|  | **Connectivity with Left Primary Visual Cortex** | |  | **Connectivity with Right Primary Visual Cortex** | |
| --- | --- | --- | --- | --- | --- |
| **Brain Region** | **Talairach Coordinates** | **Z score** |  | **Talairach Coordinates** | **Z score** |
| **DLB Mean** |  |  |  |  |  |
| Cuneus (R) | 10, -84, 13 | 5.88 |  | 8, -87, 7 | 6.23 |
| Cuneus (L) | -9, -90, 6 | 5.87 |  | -11, -85, 7 | 5.44 |
| Middle Occipital (L)  Lingual (R) | -  - | -  - |  | -18, -87, 17  12, -89, -4 | 5.40  4.85 |
| **AD Mean** |  |  |  |  |  |
| Cuneus (R) | 8, -84, 13 | 5.56 |  | 13, -74, 8 | 5.38 |
| Cuneus (L) | -3, -85, 7 | 5.66 |  | -7, -88, 7 | 5.30 |
| Precuneus (L)  Lingual (R) | -  6, -71, -2 | -  5.24 |  | -17, -48, 54  2, -84, 2 | 3.60  5.29 |
| Lingual (L)  Superior Temporal (L) | -  -36, -42, 11 | -  3.89 |  | -3, -78, -3  - | 5.01  - |
| Insula (R)  Insula (L)  Middle Temporal (L)  Caudate Tail (L)  Sub-Gyral (L)  Inferior Parietal (L)  Superior Parietal (L)  Paracentral Lobule (R)  Paracentral Lobule (L)  Postcentral (R) | -  -35, -35, 22  -49, -33, 0  -33, -38, 6  -  -  -  -  -  - | -  3.57  3.47  3.45  -  -  -  -  -  - |  | 30, -19, 25  -  -49, -35, 0  -31, -40, 11  -22, -48, 53  -31, -45, 43  -22, -62, 47  11, -34, 50  -2, -29, 56  35, -27, 46 | 3.95  -  3.28  3.64  3.70  3.48  3.32  3.41  3.02  2.96 |
| **Controls Mean** |  |  |  |  |  |
| Cuneus (R) | 12, -89, 7 | 5.67 |  | 10, -87, 7 | 5.88 |
| Cuneus (L) | -5, -82, 18 | 6.26 |  | -11, -79, 7 | 5.43 |
| Lingual (L) | 17, -58, -1 | 5.56 |  | - | - |
|  |  |  |  |  |  |
| **DLB > AD**  **DLB > Controls**  **AD > DLB**  **AD > Controls**  **Controls > DLB**  **Controls > AD** | NS differences  NS differences  NS differences  NS differences  NS differences  NS differences |  |  | NS differences  NS differences  NS differences  NS differences  NS differences  NS differences |  |

Z Statistic images were thresholded using clusters determined by z>2.3 and a corrected cluster significance of p<0.05
